# P45 - Early exclusive breastfeeding protects from preschool wheeze

**DOI:** 10.1186/2045-7022-4-S1-P100

**Published:** 2014-02-28

**Authors:** George V Guibas, Paraskevi Xepapadaki, George Moschonis, Nikolaos Douladiris, Yannis Manios, Nikolaos G Papadopoulos

**Affiliations:** 1 Allergy Department, 2nd Pediatric Clinic, University of Athens, Athens, Greece; 2 Department of Nutrition and Dietetics, Harokopio University, Athens, Greece

## Background

Thus far, exhaustive research has been conducted on a potential link of breastfeeding (BF) to wheezing illnesses. Nevertheless, conflicting evidence often emerges with several investigators reporting BF to protect from asthma, whereas others fail to show such a link; reports of an asthma-favoring role of BF can also be found. We therefore opted to explore the relation of different infantile feeding patterns with wheeze/asthma prevalence, in a cross-sectional, population-based study in preschool children.

## Methods

Wheeze ever, doctor-diagnosed asthma and perinatal data were reported via questionnaire by parents of 1871 children aged 1-5. Information on feeding practices (exclusive breastfeeding *vs* mixed *vs* formula) and their duration (two *vs* four *vs* six months) was collected. Anthropometric measurements were conducted. Logistic regression models were build in the Statistical Package for Social Sciences (SPSS version 20.0), with the wheeze/asthma variables as main outcomes. A two-tailed p value less that 0.05, was considered statistically significant.

## Results

Using the 6 months of exclusive BF as reference and following adjustment for several confounding factors (maternal prenatal smoking, maternal age at birth, gestational age, birth weight, gender, parity, passive smoking at home, parental educational level, current BMI/waist circumference and history of atopic dermatitis), we find that all regimes that did not include at least 2 months of exclusive BF were positively associated with ever wheeze (OR 1.36-2.17. 95%CI=1-3.55 p=0.001-0.044). Conversely, regimes including early exclusive BF of as low as 2 and 4 months did not positively correlate to ever wheeze (OR 0.92-1.45. 95%CI=0.53-2.88 p=0.08-0.77); Diverse BF regimes were not differentially associated with reports of doctor-diagnosed asthma (p>0.05).

## Conclusions

Early exclusive BF is associated with reduced prevalence of ever wheeze in preschoolers.

